# The Landscape of Diabetic Kidney Disease in the United States

**DOI:** 10.1007/s11892-018-0980-x

**Published:** 2018-02-19

**Authors:** O. Kenrik Duru, Tim Middleton, Mona K. Tewari, Keith Norris

**Affiliations:** 1 Department of Medicine, Division of General Internal Medicine/Health Services Research, David Geffen School of Medicine at the University of California, Los Angeles, 10940 Wilshire Blvd, Suite 700, Los Angeles, CA 90024 USA; 2 AbbVie, North Chicago, IL USA

**Keywords:** Albuminuria, Diabetes, Diabetic kidney disease, Chronic kidney disease

## Abstract

**Purpose of Review:**

The purposes of this review are to identify population characteristics of important risk factors for the development and progression of diabetic kidney disease (DKD) in the United States and to discuss barriers and opportunities to improve awareness, management, and outcomes in patients with DKD.

**Recent Findings:**

The major risk factors for the development and progression of DKD include hyperglycemia, hypertension, and albuminuria. DKD disproportionately affects minorities and individuals with low educational and socioeconomic status. Barriers to effective management of DKD include the following: (a) limited patient and healthcare provider awareness of DKD, (b) lack of timely referrals of patients to a nephrologist, (c) low patient healthcare literacy, and (d) insufficient access to healthcare and health insurance.

**Summary:**

Increased patient and physician awareness of DKD has been shown to enhance patient outcomes. Multifactorial and multidisciplinary interventions targeting multiple risk factors and patient/physician education may provide better outcomes in patients with DKD.

**Electronic supplementary material:**

The online version of this article (10.1007/s11892-018-0980-x) contains supplementary material, which is available to authorized users.

## Introduction

Diabetes mellitus is the major cause of chronic kidney disease (CKD) and end-stage renal/kidney disease (ESRD/ESKD) in the United States [1•] and globally [2]. In 2012, the estimated prevalence of diabetes (both diagnosed and undiagnosed) in the United States was 9% to 14% [3, 4]. Diabetes disproportionately affects non-Hispanic blacks (22%) and Hispanics (23%) and those with the lowest levels of education (19%) and income (18%) [4]. Type 2 diabetes mellitus (T2DM) accounts for 90% to 95% of all cases of diagnosed diabetes [3].

Approximately 25% of individuals with diabetes have diabetic kidney disease (DKD) [5•], which refers to CKD presumed to be caused by diabetes [6]. DKD is commonly diagnosed by reduced estimated glomerular filtration rate (eGFR < 60 mL/min/1.73 m^2^) and/or increased urinary albumin excretion (> 30 mg/g creatinine), a marker of kidney damage, that persist ≥3 months in the presence of longstanding diabetes and exclusion of other causes of CKD [7, 8]. In the United States, the prevalence of DKD will likely increase owing to a projected 54% increase in the prevalence of diabetes by 2030 [9].

**Fig. 1:**
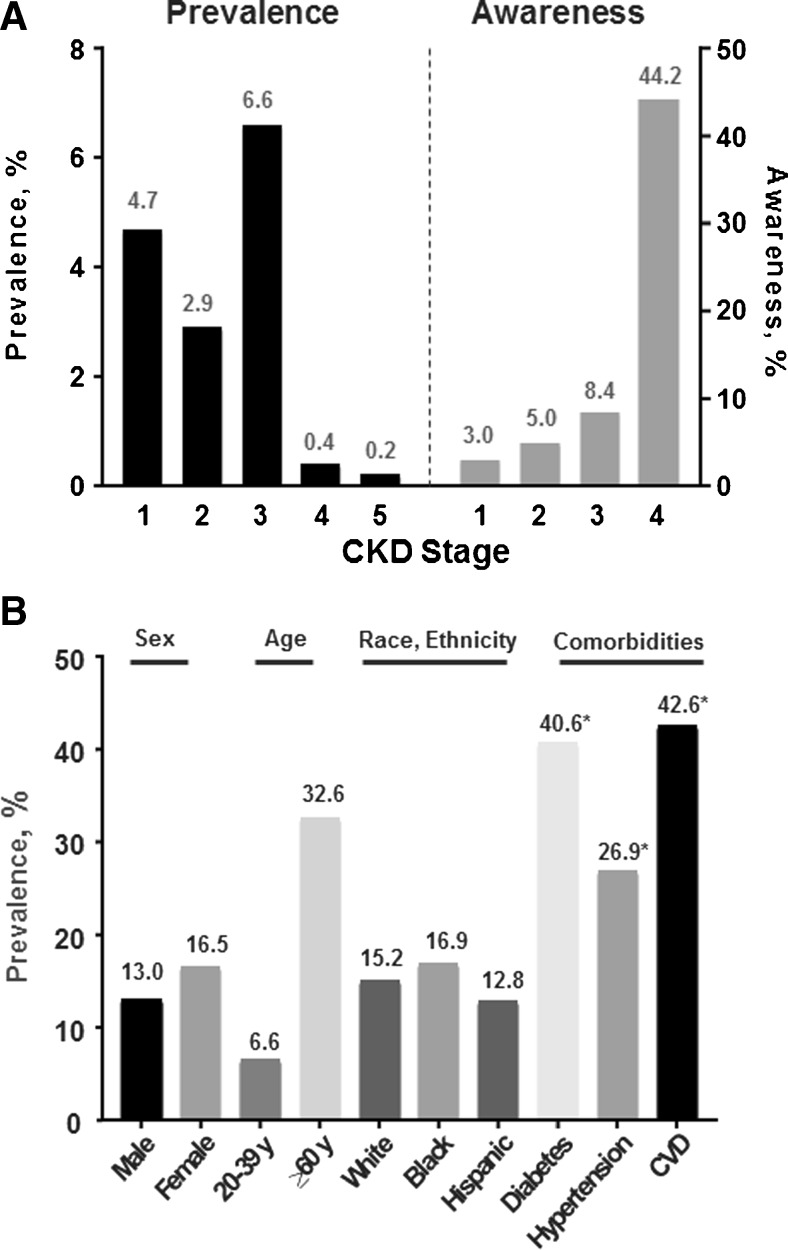
(**a**) Prevalence of CKD (2011–2014) and CKD awareness (2009–2012) by CKD stage in the NHANES population and (**b**) prevalence of CKD in the NHANES population (2011–2014) within sex, age, race/ethnicity, and risk factor categories. CKD, chronic kidney disease; NHANES, National Health and Nutrition Examination Survey. CKD stage, GFR (mL/min/1.73 m^2^): 1, > 90; 2, 60–89; 3, 30–59; 4, 15–29; 5, < 15. *Self-reported. (Data from the United States Renal Data System. 2016 USRDS annual data report: epidemiology of kidney disease in the United States. National Institutes of Health, National Institute of Diabetes and Digestive and Kidney Diseases, Bethesda, MD, 2016 [1•]. The data reported here have been supplied by the United States Renal Data System (USRDS). The interpretation and reporting of these data are the responsibility of the authors and in no way should be seen as an official policy or interpretation of the US government.)

Based on data from the United States Renal Data System (USRDS), the overall prevalence of CKD (stages 1–5) in the United States was 15% from 2011 to 2014, with stage 3 CKD being the most prevalent (Fig. 1a) [1•]. The prevalence of early CKD is generally similar across races and ethnicities (Fig. 1b). However, the prevalence of ESRD is 3.7 times greater in African Americans, 1.4 times greater in Native Americans, and 1.5 times greater in Asians than in the non-Hispanic White population; the prevalence of ESRD is almost 58% higher among Hispanics compared with non-Hispanics [1•]. The prevalence of CKD is slightly higher in women compared with men and increases with age (Fig. 1b) [1•]. However, because GFR naturally declines with age, the prevalence of actual kidney disease in the elderly may be overestimated [10, 11].

A key feature of CKD and DKD is a lack of awareness of the disease in both patients (Fig. 1a) and healthcare providers [1•, 12]. In the United States, even in patients with severe (stage 4) CKD, less than half were aware of their kidney damage [12].

The pathophysiology of DKD is typically manifested through damage to the glomerulus, interstitium, and blood vessels. At a given eGFR, higher levels of albuminuria, which are typically observed in DKD, are associated with accelerated progression to ESRD and/or decreased life expectancy [13, 14]. However, not all patients with DKD and reduced eGFR have increased albuminuria [15, 16], and some patients with albuminuria < 300 mg/mL (21% to 64%) may return to normal albumin excretion [17].

In addition to higher rates of ESRD, individuals with diabetes have an increased risk of mortality, mainly from cardiovascular disease (CVD) [18], which is strongly associated with DKD. These complications underscore the importance of screening, early detection, and treatment of DKD. Better awareness and early identification of DKD and addressing risk factors for DKD may directly impact the development and progression of the disease, helping to reduce morbidity and mortality. This review has two objectives. The first is to identify population characteristics of important risk factors for the development and progression of DKD in the United States. Second, we discuss barriers and opportunities to improve awareness, management, and outcomes in patients with DKD.

## Search Strategy

References for this review were identified through a search of PubMed for English language articles published from January 2011 to August 2017 by use of the term “diabetic kidney disease” alone and in combination with “progression” and “risk factors.” Articles pertaining to CKD and DKD epidemiology, management, albuminuria, socioeconomic factors, and patient/physician education and awareness were retrieved and reviewed. Relevant references cited in retrieved articles were also reviewed. Note, in this review, the terms African American, Black, and Non-Hispanic Black are used interchangeably based on how persons were characterized in the literature. The same holds true for the terms Hispanic and Latino.

## Consequences of CKD and DKD

In patients with CKD, the rate of decline in GFR is variable [19], and the rate of progression to ESRD and renal replacement therapy is influenced by multiple factors. Therefore, slowing the rate of progression of CKD is a priority. In an analysis of patients with CKD in a large managed healthcare organization, the proportion of patients with CKD who progressed to dialysis over a 5-year period was 1%, 1%, and 20% for CKD stages 2, 3, and 4, respectively. Mortality rates over this period were 20%, 24%, and 46% for CKD stages 2, 3, and 4, respectively [20]. Factors associated with a more rapid decline in GFR included lower serum albumin and lower hemoglobin, higher glycated hemoglobin (HbA1c), higher albuminuria, elevated blood pressure, low physical activity, and black race [19, 21, 22]. In The United Kingdom Prospective Diabetes Study of 5102 patients with newly diagnosed T2DM, 0.3%/year of patients with albuminuria 50–299 mg/L and 2%/year with albuminuria ≥ 300 mg/L progressed to ESRD over a median follow-up of 10.4 years [23]. Of note, 3%/year and 5%/year, respectively, died during this period. Thus, in patients with DKD, death is more likely than renal replacement therapy.

Chronic kidney disease is often asymptomatic, especially in its early stages. Yet, the risk for CVD is increased even in individuals with stages 1–2 CKD compared with those without CKD [24]. In an analysis of patients (*N* = 266,975) at high risk for CKD (e.g., those with hypertension, diabetes, or CVD), there was a graded and independent increased risk for all-cause and cardiovascular mortality with increasing albuminuria and decreasing eGFR below 60 mL/min/1.73 m^2^ [25]. Other studies in patients with diabetes have also found that increased albuminuria and decreased eGFR are independent risk factors for cardiovascular mortality, cardiovascular events, and renal events, including progression to ESRD [26].

## Factors Contributing to DKD

Improved communication with patients of the importance of risk factors associated with diabetes and DKD may improve management of the risk factors and the clinical outcomes. In the United States, the high prevalence of obesity (35%) [27] and metabolic syndrome (33%) [28] (three or more of increased waist circumference, hypertension, insulin resistance, or dyslipidemia) are among the major factors that contribute to the high prevalence of T2DM. Hyperglycemia and the common comorbidities of hypertension and dyslipidemia in patients with T2DM are among the major risk factors for the development and progression of DKD [29] (see Supplemental text).

The need to treat multiple risk factors increases the challenge of controlling DKD progression. An analysis of the NHANES database (2007–2010) found that only 52% of adults with diagnosed diabetes achieved HbA1c < 7%, 51% achieved blood pressure < 130/80 mmHg, and approximately 56% achieved LDL cholesterol < 100 mg/dL [30, 31]. However, the most striking feature of this analysis was that only 19% of patients achieved all three of these goals.

A more detailed discussion of these and other risk factors can be found in the online Supplement. Guidelines for target levels and treatment are briefly outlined here and in Supplemental Table 1.

### Hyperglycemia

Results from the Diabetes Control and Complications Trial (DCCT) [32] and its long-term follow-up study, Epidemiology of Diabetes Interventions and Complications (EDIC) [33] in patients with type 1 diabetes, suggested that intensive glycemic control (target HbA1c < 6%), especially early in the disease course, reduced the risk of microvascular and, to some extent, macrovascular complications and mortality. Conflicting results were observed in the Action to Control Cardiovascular Risk in Diabetes (ACCORD) trial [34], the Veterans Affairs Diabetes Trial (VADT) [35], and the Action in Diabetes and Vascular Disease: Preterax and Diamicron Modified-Release Controlled Evaluation (ADVANCE) trial [36], in which intensive glycemic control resulted in little or no significant reductions in CV outcomes in patients with T2DM and established CV disease and/or multiple CV risk factors. In ACCORD, intensive glycemic control (target HbA1c < 6%) compared with standard therapy (target HbA1c 7.0%–7.9%) was associated with an increase in mortality [34]. Subsequent analysis suggested that severe hypoglycemia was associated with an increased risk of death in both the intensive and standard therapy treatment arms of the ACCORD study [37]. Based on these studies, the American Diabetes Association (ADA) recommends that treatment goals for hyperglycemia should be individualized based on diabetes duration, life expectancy, comorbidities, risk for hypoglycemia, and patient preferences [8, 38] and target an HbA1c of 7% or lower in most patients [38, 39]. Among individuals with DKD, there is some evidence for a U-shaped relationship between HbA1c and mortality with the risk of death increasing with HbA1c < 6.5% and > 8%. Thus, a target HbA1c of ~ 7% is recommended for patients with DKD [8, 38]. Among older adults on antihyperglycemic agents, lower eGFR is associated with increased incidence of hospital encounters for hypoglycemia [40].

### Hypertension

The ADA recommends target blood pressure < 140/90 mmHg for most patients with diabetes [8, 41•], and the Kidney Disease Outcomes Quality Initiative (KDOQI) guidelines recommend < 130/80 mmHg for patients with DKD [6]. The recent 2017 American College of Cardiology/American Heart Association Task Force on Clinical Practice Guidelines also recommends a target blood pressure < 130/80 mmHg [42•]. In patients with DKD and hypertension, angiotensin-converting enzyme inhibitors (ACEIs) and angiotensin II receptor blockers (ARBs) are the recommended first-line agents for blood pressure control [6, 8].

### Dyslipidemia

Lipid abnormalities are common in patients with diabetes and typically include elevated triglycerides, reduced high-density lipoprotein (HDL) cholesterol, and elevated low-density lipoprotein (LDL) cholesterol [43]. Although specific lipid target levels are not specified, treatment guidelines recommend the use of statins to reduce the risk of cardiovascular events in patients with DKD (but not those on dialysis) [44].

### Albuminuria

Albuminuria is both a marker of kidney disease and a risk factor for CKD progression and cardiovascular events [7]. It is important to note that there is a graded association of albuminuria and risk for progression to ESRD, CVD, or death with increasing risk as levels of albuminuria rise [25, 38, 45, 46].

## Diagnosis of DKD

As noted earlier, DKD is commonly diagnosed by an eGFR < 60 mL/min/1.73 m^2^ and/or albuminuria > 30 mg/g creatinine in the presence of longstanding diabetes and no other causes of CKD [7, 8]. However, some patients with diabetes develop DKD without an increase in urinary protein excretion, whereas remission to normal protein excretion has been reported in some patients with DKD [17]. Therefore, whether or not moderate levels of albuminuria by itself are an early predictor of DKD and its progression remains controversial [17, 47, 48]. This may be due to multiple pathophysiological factors which are part of the constellation of DKD (interstitial damage, vascular and/or glomerular) [49] and reinforces the need to search for more sensitive and specific biomarkers [50, 51]. Nevertheless, albuminuria, together with eGFR, remains useful for monitoring kidney function [38, 52]. The current lack of awareness of the significance of DKD has contributed to underuse of these diagnostic tools. A summary of the current state of screening for reduced kidney function may be found in the Supplement.

## Barriers to DKD Management

A key problem associated with CKD and DKD is a lack of awareness of the disease (Fig. 1a).

**Fig. 2:**
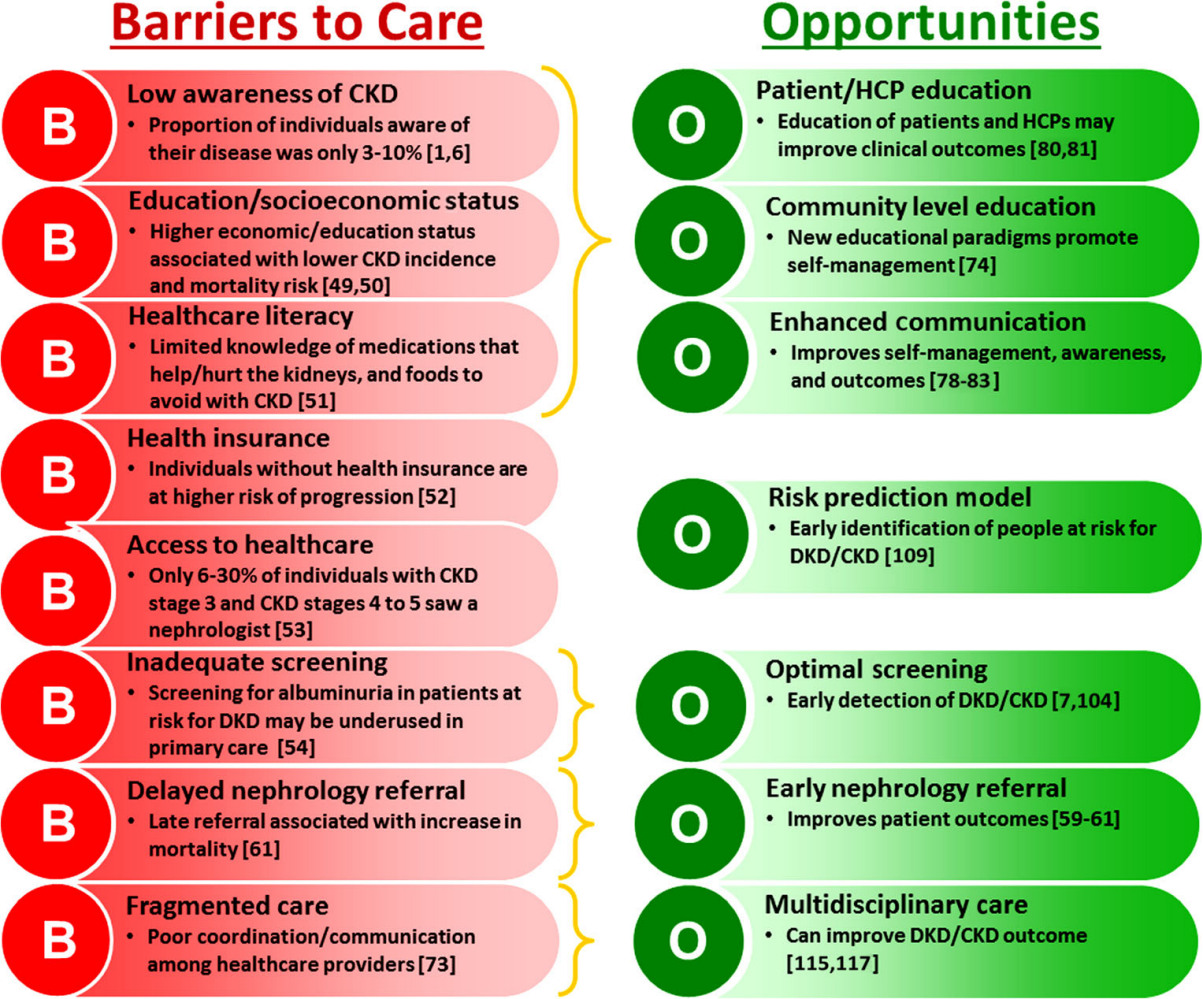
Barriers to care and opportunities to improve awareness, management, and outcomes in patients with DKD

Effective management of DKD is multidisciplinary and requires heightened awareness of DKD by healthcare providers and patients as well as effective communication and collaboration between healthcare providers, specialists, and their patients. Additional barriers exist affecting the successful management of DKD, including socioeconomic factors, access to healthcare and health insurance, and education and awareness of DKD. Unfortunately, populations at highest risk of T2DM, DKD, and ESRD (e.g., African Americans, Native Americans, Hispanic/Mexican Americans, and those with low socioeconomic status) [1•, 4] are also those that have the most barriers to overcome in accessing quality healthcare [53, 54]. Figure 2 presents a summary of some of the barriers to effective care and opportunities for minimizing those barriers.

### Patient-Specific Barriers

#### DKD Awareness

Data related to patient-specific barriers for DKD are limited; thus, we present data for overall CKD, recognizing that these data should be applicable to DKD as well. Data supporting the discussion of patient-specific barriers can be found in Supplemental Table 2. Awareness of CKD in general and DKD in particular is low in the United States (Fig. 1a) [1•, 55] and globally [56•]. Patient awareness of CKD is low at all stages of CKD, even stage 4 [55], but does increase with the severity of CKD [57, 58], in the presence of or risk for comorbidities (e.g., diabetes, hypertension, obesity, CVD) [56•, 57–59], and increasing laboratory markers of CKD complications (e.g., albuminuria, hyperkalemia, hypophosphatemia, anemia, acidosis, elevated blood urea nitrogen) [60].

The greater awareness of CKD among men may be because men normally have higher serum creatinine than women, and physicians may view them as being at higher risk for CKD than women [57]. However, this could also reflect provider biases in patient communication. A greater awareness among African Americans than non-Hispanic White or Mexican Americans may be due to increased recognition by African Americans and their physicians of race as a powerful risk factor for ESRD, and/or the greater family history of CKD/ESRD in this population [57].

#### Education and Socioeconomic Status

Both the extent of educational attainment and socioeconomic status have been shown to be associated with barriers to care in CKD patients. A variety of studies have demonstrated that income and level of education are associated with risk factors for and progression of CKD [61, 62]. Because level of education and socioeconomic status are not uniformly distributed across ethnic groups, the effects of limited education and socioeconomic status are more acute in racial and ethnic minority populations.

#### Healthcare Literacy

In general, poor health literacy (skills needed to function effectively in the healthcare environment) is associated with more hospitalizations and emergency room use, lower use of preventative services, and poorer adherence to medications, and may partially explain racial disparities in healthcare service utilization and outcomes [63].

#### Access to Health Insurance and Healthcare

In the United States, individuals with CKD and no health insurance were less likely to receive treatment for risk factors associated with the progression of CKD, such as hypertension, diabetes, and obesity, than individuals with CKD and health insurance [64]. A smaller proportion of African American and Hispanic individuals with CKD reported having a physician than non-Hispanic White individuals [65].

### Provider-Specific Barriers

#### DKD Awareness

Primary care physicians appear to have a low awareness of CKD and DKD. In a multicenter observational study in 466 primary care practices, only 47% of primary care providers (PCPs) identified CKD in their patients with T2DM, primarily because of underutilization of screening assessments, such as urinary albumin/protein excretion [12]. Importantly, patient awareness of DKD was 81% in patients in whom a PCP diagnosed DKD compared with only 3% in the absence of a DKD diagnosis, suggesting a link between clinician diagnosis of DKD and patient awareness [12]. Data supporting the discussion of provider-specific barriers can be found in Supplemental Table 3.

Some studies suggest that screening for albuminuria in patients at risk for DKD may be underused in primary care [12, 66]. Unsurprisingly, family practice physicians and general internists were less aware of clinical practice guidelines for CKD than nephrologists [67]. Furthermore, in another survey conducted in predominantly African American communities, most PCPs recognized diabetes and hypertension as risk factors for CKD; however, 34% did not consider family history of CKD, and 22% did not consider race as a risk factor for CKD [68].

#### Contact Time and Communication with Patient

Diabetes care professionals commonly cite lack of available time as a barrier for effective communication with patients [69]. The introduction of electronic health records (EHR) has led to physicians spending almost 2 h on computer and clerical work for every hour they spend with patients [70]. Unfortunately, this is likely to worsen before it becomes better.

#### Need for Early Referral to a Nephrologist

In a prospective cohort study of patients from primary care, referral of patients with T2DM and early DKD (defined as albuminuria 30–300 mg/g creatinine or eGFR 60–89 mL/min/1.73 m^2^) to a nephrologist was associated with better preservation of renal function and better control of blood pressure, when compared with continued treatment by PCPs alone [71]. Additional studies have shown that referral of patients with CKD to nephrologists slows the decline in GFR and reduces mortality [72, 73].

The finding that nephrologists deliver better CKD-related care to patients with early CKD than PCPs is not unexpected, but there is not a sufficient nephrology workforce in the United States [74] and globally [75] to care for all patients with CKD, and there are many other aspects of total patient care that PCPs do better than nephrologists. Therefore, on balance, education on occasional early consultation for diagnosis and treatment, and timely referral for management support in all patients with advanced CKD stages is critical. Recently, a panel of internists and nephrologists developed a practical approach for the Kidney Disease Outcomes Quality Initiative and recommended referral to nephrology specialists when eGFR fell below 30 mL/min/1.73 m^2^, or for severe albuminuria (≥ 300 mg/g creatinine) [76] or acute kidney injury [76, 77].

The critical need for referral is further highlighted by a report from Gillespie et al. [78] who examined Medicare data from 2006 to 2010 and found that 33% of patients received no nephrology care before the onset of ESRD. According to an analysis of the USRDS database (2007–2012), poverty, African American race, and Hispanic ethnicity were independently associated with lower rates of pre-ESRD nephrology care [79].

### Treatment-Specific Barriers

Treatment of diabetes and CKD is complex, both for the provider and patient. Clinical inertia, or the failure to intensify treatment when a patient is not at recommended goals, is of particular concern. In a retrospective analysis of people with T2DM, some patients remained in poor glycemic control for up to 7 years before intensification of treatment [80]. Similarly, in an analysis of patients with CKD and uncontrolled hypertension, the prevalence of clinical inertia was 44% [81]. Moreover, certain classes of medications, such as ACEIs, ARBs, and statins, may be underutilized in patients with CKD and comorbidities [82].

Patients with CKD usually have multiple comorbidities and may be prescribed more than 20 medications [82]. Thus, suboptimal medication adherence represents a substantial barrier to effective management of CKD. In a study of patients with CKD and hypertension, only two thirds (69%) of patients reported appropriate medication adherence; older age and higher income were associated with higher adherence [83]. However, it should also be noted that patient self-reporting may overestimate medication adherence [84].

### Health System–Specific Barriers

The first barrier to overcome for optimal DKD care is to ensure the understanding of the significance and limitations of appropriate laboratory testing [85]. Additional health system barriers include poor coordination of a broad range of providers across diverse settings, including inpatient and outpatient facilities, emergency departments, pharmacies, and dialysis facilities, that limit the provision of accurate and timely information [86]. The introduction of EHRs addresses some but not all of the barriers of a distributed healthcare system because many system components do not interact [86]. Other factors such as delivery system design, provider decision support tools, integration of kidney disease management into existing diabetes care processes, and the creation of “medical neighborhoods” formed through accountable care organizations are also needed to improve CKD care [86, 87].

### Community-Specific Barriers

Multiple barriers to DKD care exist at the community level, especially in high-risk communities, and include low health literacy, being un- or under-insured, difficulty in accessing quality care, limited availability of CKD information, lack of readiness to learn, lack of trust in the health system, and other factors [54, 88, 89]. Recent approaches using new educational paradigms and new groups of health professionals have demonstrated great potential to promote self-management, and others should be considered, such as the use of lay health educators and engagement of community-based and allied health professionals in early CKD management [87]. Family or close friend/confidant-based interventions can assist in implementing and increasing adherence to lifestyle, nutritional, and pharmacologic interventions [87]. Because health literacy, educational attainment, and cultural beliefs and behaviors vary widely across different communities, efforts to adapt and assess the effectiveness of many existing educational materials to address the needs of diverse populations with CKD or the use of novel strategies such as social or digital media are needed [87, 88, 90].

## Recommendations for Better DKD Management

### Patient and Physician Education

The reviewed literature herein suggests that patients’ and healthcare providers’ knowledge and awareness of CKD and DKD can be improved. Many patients with CKD or ESRD do not feel engaged (limited knowledge of their disease, complex treatments), and many do not receive patient-centered care [91]. Healthcare providers may not recognize CKD in its early stages and may not be familiar with clinical practice guidelines or recommendations [67].

Educational efforts aimed at patients and healthcare providers have been shown to improve clinical outcomes. For example, brief physician-led educational discussions with patients improve their CKD awareness and understanding of the severity of their disease [92]. Many patients want to be educated about CKD [88], which may improve blood pressure [93, 94], lengthen time to dialysis/transplantation [95], and improve survival [93, 94]. Educating individuals with progressive CKD about kidney function, kidney disease, diet, and lifestyle changes delayed the initiation of dialysis [95] and prolonged survival [94]. Moreover, in patients with diabetes, training sessions about health literacy and numeracy were associated with better glycemic control and diabetes self-management compared with patients who did not receive such training [96]. Similarly, in a Canadian study of targeted screening for CKD [97], in which individual counseling was provided for those at risk for CKD, most participants (90%) reported health behavior changes in a post-screening survey, including dietary changes (80%), better adherence to recommendations from their healthcare providers (66%), and making lifestyle changes (increasing activity, reducing stress, or weight loss) (76%).

The burden of PCP workload superimposed with patient struggles with inadequate health literacy, coping with new diagnoses, and implications of a possible poor prognosis conspire to limit effective communication. There are tools currently available to help foster such communication. Educational programs are available for patients (National Kidney Disease Education Program) [98], and they are a covered benefit under Medicare for patients with stage 4 CKD [99]. A multidimensional support program (disease knowledge, self-management, and motivation skills) has been shown to improve HbA1c levels, albuminuria, and physical activity in patients with DKD [100]. CKD educational tools for physicians are available from various organizations, including the National Kidney Foundation [101], the Medical Education Institute [102], and the Renal Physicians Association [103].

### Optimal Screening

The United States Preventative Services Task Force concluded that there was insufficient evidence to assess the benefits and harms of screening for CKD in asymptomatic adults [104]. By contrast the American College of Physicians recommends against screening for CKD in asymptomatic adults without risk factors for CKD [105], while the American Society of Nephrology recommends that all adults undergo screening for CKD [106].

Concerns about total population screening include inappropriate disease labeling impacting insurability as well as false-positive results with attendant unnecessary testing and treatment. Also, false-negative results create an unwarranted sense of assurance and can delay needed interventions when the actual disease is present [104, 105].

Modeling and other studies have generally found that screening for CKD may be cost-effective only in non-Hispanic Blacks [107] or in older patients with diabetes and/or hypertension or at longer intervals of 5–10 years [108–110]. However, a recent simulation study using CKD risk scores based on diabetes, hypertension, anemia, and CVD suggested that CKD screening may be cost-effective in a broader population [111]. To our knowledge, no randomized trials have evaluated the effectiveness of screening for CKD to improve patient care or outcomes.

Current guidelines from the ADA [112] and the National Kidney Foundation [6] recommend that patients with T2DM be screened annually for albuminuria and eGFR (Supplemental Table 4). Albuminuria in the range of 30–300 mg/g creatinine is best measured by a spot urine sample from the first morning void and should be confirmed with two additional measurements during the next 3 to 6 months [6]. If an albuminuria test is not available, a reagent strip may be used [6], preferably a more sensitive strip that can detect lower levels of albuminuria. The use of select microalbuminuria detection strips can provide results similar to the actual albumin:creatinine ratio [113]. In primary care settings, improved screening rates for microalbuminuria in patients with diabetes can be achieved by quality improvement processes that include education of staff on clinical practice guidelines [66]. In addition to diabetes, population characteristics of persons at increased risk for whom screening for CKD should be considered include those with hypertension, family history of CKD, low socioeconomic status, the elderly, and high-risk racial/ethnic groups [54, 114–119].

In patients with known DKD, eGFR should be monitored more frequently; every 6 months if eGFR is 45–60 mL/min/1.73 m^2^ and every 3 months if eGFR is 30–44 mL/min/1.73 m^2^ [8]. Serum creatinine can be measured in a spot blood sample and eGFR estimated using various equations [120] either by a clinical laboratory or by online eGFR calculators [66], although most clinical labs now automatically report eGFR compared with less than 50% only 10 years ago [121].

### Risk Prediction Models

Early detection and treatment of DKD delays progression to ESRD [122]. Methods to identify individuals at risk for developing DKD or at risk for progression to ESRD are important because most patients with DKD are initially identified by their PCPs [123]. A number of models have been developed and validated that predict the risk for the development and/or progression of CKD and DKD using readily available laboratory values, such as eGFR, urinary albumin excretion, and blood pressure, as well as age, sex, diabetes status, and ethnicity [124]. Overall, the most predictive variables appear to be eGFR and urinary albumin excretion [125–127]. The development of renal risk scores is still in an early stage, and most models have limitations [123, 124]. Additional research focused on identification and validation of novel biomarkers for DKD prediction is ongoing [128, 129].

### Multidisciplinary Care

Owing to the complex nature of DKD and associated comorbidities, the care of patients with DKD is often divided among PCPs, specialists, nurses, and other healthcare providers. Multidisciplinary care consisting of an integrated team of physicians, nurses, dietitians, and educators has been shown to slow the decline in kidney function and the progression to ESRD [130]. For example, in a group of patients with stage 3 CKD and diabetes and/or hypertension, multidisciplinary care with a team composed of a PCP, nephrologist, pharmacy specialist, diabetes educator, dietitian, social worker, and nephrology nurse resulted in an annual decline in eGFR that was approximately half of that observed in patients with usual care (a PCP and nephrologist referral) [131]. In another study of patients with stages 3–5 CKD (44% with diabetes), multidisciplinary care slowed eGFR decline, decreased cardiovascular events and infections, reduced the need for renal replacement therapy, and was more cost-effective than usual care (nephrology outpatient clinic) [132].

As noted earlier, a multidimensional educational support program can improve HbA1c levels, albuminuria, and physical activity in patients with DKD [100]. Similarly, intensive management of patients with diabetes consisting of online health coaching, individualized nutrition education to reduce carbohydrate intake, and behavioral support for 10 weeks improved glycemic control, enhanced weight loss, and reduced the number and/or dosage of antidiabetes medications [133]. Remotely delivered intensive behavioral counseling programs may also be effective and have been associated with considerably reduced medical expenditures [134].

Treatment guidelines and pharmacotherapy recommendations for diabetes, DKD, and CKD are beyond the scope of this review but can be found in publications from the ADA [112] and National Kidney Foundation [6, 38, 39, 135••]. It appears, however, that multifactorial and multidisciplinary intervention targeting multiple risk factors may provide better patient outcomes. For example, in the STENO-2 trial in patients with T2DM, diet and exercise together with therapy targeting hyperglycemia, hypertension, and dyslipidemia; smoking cessation; aspirin; and antioxidants significantly reduced the risk for the development of nephropathy compared with conventional therapy [136]. After 21 years of follow-up, the multifactorial therapy group had reduced progression to macroalbuminuria, a slower rate of decline in eGFR, and a trend toward less progression to ESRD compared with the conventional therapy group [137••].

## Conclusion

Diabetic kidney disease remains a major healthcare issue. Awareness of DKD is low among both patients and their healthcare providers. Although many barriers exist, diagnostic tools needed to increase awareness are readily available, and programs that increase disease awareness have been demonstrated to be both clinically effective and effective in reducing the use of healthcare resources. DKD disproportionately affects minorities, and those with more limited education, lower socioeconomic status, and reduced access to healthcare and health insurance. Progressive policy changes are needed to address these social determinants of health.

## Strategies to Improve DKD Outcomes

Given the strong association of increasing levels of albuminuria and clinical outcomes, albuminuria should be reported as a continuous variable, rather than the more limited terms microalbuminuria and macroalbuminuria. This will promote more attention at the health system and provider levels to each patient’s risk status and can help to guide response to therapy. Novel strategies to improve DKD outcomes (Table 1, Fig. 2) include improved targeting of high-risk patients and enhanced communication and education through the use of tele-health technology to both obtain patients’ vital signs and deliver kidney health services to expand choice, facilitate access to care, and deliver patient-centered kidney specialty care services and education via synchronous and asynchronous approaches [138]. Improved delivery of quality healthcare can be facilitated by the use of CKD clinical decision support tools to promote timely referral [139], provider incentives, interdisciplinary care models [88], and the use of CKD patient navigators, especially for those with advanced disease and approaching ESRD to help coordinate care, address system barriers, and educate/motivate patients [140]. Additional considerations include promotion of self-management support, shared decision making, use of digital media, and family and community engagement [88]. Finally, promoting policies to support public awareness and patient education programs requires ongoing advocacy.

**Table 1:** Strategies to improve diabetic kidney disease outcomes: a call to action

Improve targeting of high-risk patients	Enhance communication and education	Improve delivery of quality healthcare
Use of electronic health records to identify high-risk persons and alert providers	Enhanced patient-centered approaches	Use of DKD patient navigators as ESRD approaches
Small area analysis to identify communities with higher rates of DKD/ESRD	Community-level education strategies to increase awareness of and impact on DKD risk factors	Advocacy for single-payer and/or disease- management health care systems
Increased nephrology referral of patients with stage 3/4 DKD	Increased patient input into DKD care strategies	Increased system level support for PCPs

DKD, diabetic kidney disease; ESRD, end-stage kidney disease; PCP, primary care provider

## Electronic supplementary material

ESM 1 (DOCX 176 kb)
