# Supplementary material for: The Landscape of Diabetic Kidney Disease in the United States
Source: Curr Diab Rep. 2018 Feb 19;18(3):14. doi: 10.1007/s11892-018-0980-x (PMC5817078; doi:10.1007/s11892-018-0980-x)
Supplement: Supplementary file 1 — (DOCX 176 kb) [file 11892_2018_980_MOESM1_ESM.docx]

**Supplement for The Landscape of Diabetic Kidney Disease in the United States**

**Factors Contributing to Diabetic Kidney Disease**

Awareness of diabetes and CKD risk factors among the US population is low [1,2]. Hyperglycemia and the common comorbidities of hypertension and dyslipidemia in patients with T2DM are among the major risk factors for the development and progression of DKD [3]. In addition, several other factors have been identified as contributing to CKD or DKD progression.

*Hyperglycemia*

Hyperglycemia is an important factor in the initiation and progression of DKD. Poor glycemic control is independently associated with CKD in individuals with diabetes[4]. Numerous clinical trials in patients with T2DM have demonstrated that effective glycemic control (HbA1c of approximately 7%) reduces the development of albuminuria and improves renal outcomes (eg, doubling of plasma creatinine, need for renal replacement therapy) [5-9]. A reduced risk of 15% to 32% in mortality has been observed with some of the newer anti-diabetes medications in patients with T2DM and high cardiovascular risk [10,11].

Treatment goals for hyperglycemia are based on diabetes duration, life expectancy, comorbidities, risk for hypoglycemia, and patient preferences [12,13]. Current clinical practice guidelines recommend a target HbA1c of approximately 7% in most patients to prevent or delay the progression of diabetes complications, including DKD [13,14]. In some patients with DKD and comorbidities, a target HbA1c >7% may be more appropriate [13,15].

*Hypertension*

The American Diabetes Association (ADA) recommends target blood pressure <140/90 mmHg [15] and the Kidney Disease Outcomes Quality Initiative (KDOQI) guidelines recommend <130/80 mmHg for patients with DKD [16]. In the 2007 to 2010 NHANES population, only 51% of adult patients with diagnosed diabetes achieved blood pressure <130/80 mmHg and 72% achieved <140/90 mmHg [17,18]. In patients with DKD and hypertension, angiotensin-converting enzyme inhibitors (ACEIs) and angiotensin II receptor blockers (ARBs) are the recommended first-line agents for blood pressure control [15,16]. The beneficial effects of ACEIs or ARBs in reducing the development of DKD [19] or slowing the progression of DKD [20,21] were demonstrated in randomized, placebo-controlled trials in patients with diabetes. While the mechanism is not fully understood, some effects of ACEIs or ARBs on DKD appear to be independent of their blood pressure‒lowering effects [19-22].

*Dyslipidemia*

Lipid abnormalities are common in patients with diabetes and typically include elevated triglycerides, reduced high-density lipoprotein (HDL) cholesterol, and elevated low-density lipoprotein (LDL) cholesterol [23]. Analysis of data from the ADVANCE trial found that in patients with T2DM, lower baseline HDL cholesterol was a significant and independent predictor of the development and progression of DKD [24]. In addition, a retrospective analysis of 15,362 patients with T2DM found that elevated triglycerides (≥150 mg/dL) and low HDL cholesterol (<40 mg/dL in men and <50 mg/dL in women) were independent risk factors for the development of DKD over 4 years [25].

*Albuminuria*

Albuminuria should be viewed as a continuous variable and reported as such, instead of using the more traditional categorical terms microalbuminuria and macroalbuminuria. Filtration of large amounts of protein may activate inflammatory pathways within the kidney, resulting in interstitial fibrosis, and may increase progression to ESRD [26]. Persistent microalbuminuria (30‒300 mg/day) over the course of 3–6 months is the most commonly used clinical marker of glomerular damage and is often the earliest sign of DKD[27]. In individuals with CKD, increased albuminuria and a lower eGFR are independently associated with all-cause mortality, cardiovascular mortality, and progression to ESRD [28-30]. In post hoc analyses of randomized clinical trials in patients with T2DM and DKD, reduction of albuminuria with an ARB was associated with a reduction in a composite endpoint of doubling of serum creatinine, ESRD, or death [31] or with a slower rate of decline in eGFR [32].

*Additional or Less-studied Factors*

In addition to hyperglycemia, hypertension, and dyslipidemia, obesity, especially abdominal obesity [33], is becoming more widely recognized as a CKD/DKD risk factor with major public health implications [34]. Obesity can act in concert with hyperglycemia and hypertension to promote glomerular hyperfiltration, increased production of growth factors and adipokines, which may lead to fibrosis, glomerulosclerosis, CKD progression, and ultimately ESRD, and premature cardiovascular events [34-36].

Acute kidney injury (AKI) is defined as an abrupt decrease in kidney function and may be caused by many factors, including volume depletion, surgery, sepsis, radiocontrast agents, and nephrotoxic drugs [37]. Regardless of the initiating event, AKI may lead to subsequent CKD [38], even in patients with mild AKI whose renal function recovers within 2 days [39]. AKI independently increases the risk of long-term mortality [40]. In hospitalized patients with diabetes, AKI was a risk factor for stage 4 CKD and each additional episode of AKI doubled the risk of stage 4 CKD [41].

Less compelling evidence suggests that nontraditional risk factors such as uric acid [42], systemic inflammation [43,44], and oxidative stress [43], may be involved in the development and progression of CKD or DKD.

*Combination of Risk Factors*

Hyperglycemia, Hypertension, and Dyslipidemia

Despite the recognized importance of hyperglycemia, hypertension and dyslipidemia in the development and progression of DKD, an analyses of the NHANES database (2007‒2010) found that only 52% of adult patients with diagnosed diabetes achieved HbA1c <7%, 51% achieved blood pressure <130/80 mmHg and 72% achieved <140/90 mmHg [17,18]. Furthermore, approximately 56% achieved LDL cholesterol <100 mg/dL. Only 19% of patients achieved all 3 goals of HbA1c <7%, blood pressure <130/80 mmHg, and LDL cholesterol <100 mg/dL; meeting all 3 goals was more common in non-Hispanic White than in Mexican American patients and all Hispanic patients [18].

*Underuse of screening limits achievement of target goals for risk factors*

Early intervention and aggressive treatment of DKD is essential to slow the progression to ESRD. For example, an analysis of 4 clinical trials that assessed the effects of inhibition of the renin-angiotensin system (RAS) in patients with T2D found that RAS inhibition, compared with placebo, in the earliest stages of DKD (eGFR >60 mL/min/1.73 m^2^ and albumin:creatinine ratio <30 mg/g) delayed the predicted progression to ESRD (defined as the need for renal replacement therapy) by 4.2 years compared with intervention at an intermediate stage (eGFR 30–60 mL/min/1.73 m^2^), 3.6 years delay, or at a late stage (eGFR < 30 mL/min/1.73 m^2^), 1.4 years delay [45]. Current guidelines from the ADA [15] and National Kidney Foundation [16] recommend that patients with T2DM be screened annually for albuminuria and eGFR and more frequently as eGFR decreases. Yet, according to the USRDS, among patients with diabetes, only 38% were tested for urine albumin and even a lower proportion of non-diabetic patients with hypertension (6%) were tested from 2000 to 2013 [46]. Among patients with both diabetes and hypertension, 91% had serum creatinine testing, but only 40% had urine albumin testing. The difference in creatinine versus urine albumin testing in the latter group of patients may be because serum creatinine is usually included in a standard panel of tests, whereas urine albumin must be ordered separately [46].

In 2013, in Medicare patients (≥65 years of age) without a diagnosis of CKD, 78% received serum creatinine testing and 12% were tested for urine albumin [46]. In contrast, in patients with a diagnosis of CKD, only 23% were tested for urine albumin the year following diagnosis if they visited a PCP versus 50% if they visited a nephrologist; patients with CKD were more likely to visit a PCP (91%) than a nephrologist (30%), even those patients with stage 4 CKD (84% vs 67%). More than 90% received serum creatinine testing, regardless of PCP or nephrologist visit [46].

**Supplemental Table 1.** **Clinical Practice Guideline Goals for the Treatment of DKD and Comorbidities**

| American Diabetes Association [12] | - HbA1c   - <7% in most patients   - >7% in patients with comorbidities - BP   - <140/90 mmHg   - <130/80 mmHg in patients with albuminuria and risk of CVD and DKD progression   - ACEIs and ARBs are recommended for most patients |
| --- | --- |
| KDOQI [13,16] | - HbA1c   - ~7.0%   - >7.0% in patients with comorbidities, limited life expectancy, or risk of hypoglycemia - BP   - <130/80 mmHg   - ACEIs and ARBs ± a diuretic are recommended for most patients - Lipids   - Lower LDL-C with statins or statin/ezetimibe   - Statin therapy not recommended for patients on dialysis |

ACEI, angiotensin-converting enzyme inhibitor; ARB, angiotensin receptor blocker; BP, blood pressure; CVD, cardiovascular disease; DKD, diabetic kidney disease; HbA1c, glycated hemoglobin; KDOQI, Kidney Disease Outcomes Quality Initiative; LDL-C, low density lipoprotein cholesterol.**Supplemental Table 2. Data identifying patient-related barriers to DKD management**

| DKD awareness | - In adults with CKD in the NHANES database (2009–2012) [46] and the Kidney Early Evaluation Program (KEEP)(2000–2009), a community-based screening program [47], the overall proportion of individuals aware of their disease was only 3% to 10%; awareness was especially low in the early stages of CKD: stage 1, 3% to 5%; stage 2, 5% to 7%; stage 3, 8% to 10%; stage 4, 39% to 44%. - Men (age, ≥20 years) in the NHANES database, were twice as likely to be aware of CKD as women [48]. - African American patients (19%) were more aware of CKD than non-Hispanic White (7%) or Mexican American (12%) patients [48]. - In 6 world regions, including eastern Asia, Africa, Eastern Europe, Middle East, and Latin America, patient awareness of CKD was 6% in the general population and 10% in high-risk populations (patients with a diagnosis of CKD, hypertension, diabetes, or CVD) [49]. |
| --- | --- |
| Education, Socioeconomic status | - In a single-site, longitudinal, population-based cohort study of African Americans in Jackson, Mississippi, 18% of whom had diabetes, higher socioeconomic status was associated with a lower risk for CKD and CKD risk factors compared with a lower socioeconomic status. Individuals with high income were 41% less likely to have CKD than individuals with lower income [50]. - Among the US KEEP participants (29% with diabetes), higher education level (completed college or more) was associated with a 24% lower mortality risk compared with a lower education level (completed high school or less) [51]. - The Study of Heart and Renal Protection (SHARP,) conducted with participants from 18 countries with moderate-to-severe CKD (n=9,270), there were significant unadjusted trends for increased vascular events (eg, myocardial infarction, stroke, heart failure) and increased mortality with lower educational attainment [52]. Compared with participants with college and postgraduate education, all-cause mortality was twice as high in those without formal education. There was no association between educational level and progression to ESRD or doubling of serum creatinine levels. - In an analysis of 15,353 patients with CKD stages 3–5 in the San Francisco Department of Public Health's Community Health Network that serves the urban poor, 72% of patients were racial or ethnic minorities, 73% earned less than $15,000/year, and 46% were unemployed, disabled, and/or were receiving public assistance. The rate of progression to ESRD was over 2-fold higher among non-Hispanic Blacks compared with other racial-ethnic groups, and Hispanics and Asians/Pacific Islanders progressed to ESRD at approximately twice the rate of non-Hispanic Whites [53]. - Among 22,828 participants in the Reasons for Geographic and Racial Differences in Stroke (REGARDS) study who had urine albumin measurements, ACR was higher among non-Hispanic Blacks than non-Hispanic Whites and lower income was associated with a higher prevalence of albuminuria (ACR > 30 mg/g) in both races. After adjusting for confounding factors, decreasing income was independently associated with increased ACR only among non-Hispanic Blacks [54]. |
|  |  |
| Healthcare Literacy | - In the Chronic Renal Insufficiency Cohort study, which is an ongoing prospective cohort study of adults with mild-to-moderate CKD from 7 US clinical centers, the proportion of patients with limited health literacy (reading comprehension) was 16% overall and more prevalent among non-Hispanic Blacks (28%) versus non-Hispanic Whites (5%). Limited health literacy was associated with lower income and education, lower eGFR, and a self-reported history of CVD [55]. - In a survey of 406 patients with CKD recruited from a nephrology clinic, only 19% understood the relationship between proteinuria and poor kidney function, 40% understood the role of the kidney in glucose homeostasis, and 22% were unaware that CKD may be asymptomatic [56]. - In a survey of general kidney knowledge in the same population of patients, 72% of the patients had little or no knowledge about medications that may help the kidney, 63% were unaware of medications that may hurt the kidney, 61% had little or no knowledge about foods to avoid with CKD, and 51% had little knowledge of kidney function [57]. |
| Access to Health Insurance | - An analysis of 86,588 US KEEP participants (<65 years of age) found that individuals without health insurance (28%) were 82% more likely to die and 72% more likely to begin renal replacement therapy than those with private insurance [58], even after adjustment for demographic differences. Individuals without health insurance were more likely to be younger, have ≤12 years of education, and be Hispanic. - In the US KEEP population (2000‒2010) [59], the proportion of individuals who saw a nephrologist (with or without a generalist) was <6% for those with CKD stage 3 and <30% with CKD stages 4 to 5. Compared with non-Hispanic White and African American participants, Hispanic participants perceived a greater difficulty in obtaining medical care across all stages of CKD. In the early stages of CKD (stages 1‒2), more African Americans (13%) and Hispanic (18%) individuals reported not having a physician than did non-Hispanic White (9%) participants. In addition, non-Hispanic White participants were more likely to have health insurance and medication coverage (81% and 55%, respectively) than African Americans (75%, 48%) and Hispanics (54%, 40%). |

**Supplemental Table 3. Data identifying health care provider-related barriers to DKD management**

| DKD awareness | - In an observational study of 466 primary care practices in the United States, only 12% of patients with T2DM with DKD were identified as having DKD by their PCPs [60]. - An analysis of over 10,000 patients in a managed care organization with a baseline eGFR of 10 to 60 mL/min/1.73 m^2^ found that only 14% had a CKD diagnosis at baseline [61]. - A national survey of PCPs and nephrologists found that PCPs, compared with nephrologists, were less likely to recognize a hypothetical patient with CKD (family practice physicians, 56%; general internists, 71%; vs nephrologists, 96%) and were less likely (71%–74%) to recommend referral of the hypothetical patient to a nephrologist than were nephrologists (96%) [62]. |
| --- | --- |
| Inadequate screening | - Among patients with diabetes in the USRDS database from 2000 to 2013, only 38% were tested for urine albumin and even a lower proportion of non-diabetic patients with hypertension (6%) were screened for urine albumin [46]. |
| Contact Time and Communication With Patient | - In a study of primary care clinics, the median time spent on discussing diabetes self-care activities with patients was 5.2 minutes (range, 1 to 17 minutes), which represented only 24% of the total visit time [63]. |
| Need for Early Referral to a Nephrologist | - In a systematic review of 40 longitudinal cohort studies of 63,887 patients with CKD, early referral (more than 1‒6 months before dialysis) to a nephrologist was associated with a reduction in mortality and hospitalization compared with patients with late referral (less than 1‒6 months before dialysis) [64]. - In a Canadian study of outpatients with CKD, cumulative care (number of visits) and the consistency of care in the 3 to 6 months before renal replacement therapy was associated with a reduced 1-year mortality and a longer interval before starting renal replacement therapy [65] - Analysis of Medicare data from 2006 to 2010 showed that 33% of patients received no nephrology care before the onset of ESRD. Pre-ESRD nephrology care was reported to be lowest among African Americans (38%), Native Americans (36%), and Hispanics (40%) and among those with no health insurance (60%). Pre-ESRD nephrology care was most common in New England and western states [66]. - According to an analysis of the USRDS database (2007‒2012), poverty, African American race, and Hispanic ethnicity were independently associated with lower rates of pre-ESRD nephrology care [67]. - In the USRDS database, among Medicare patients (≥65 years of age) with a diagnosis of CKD in 2012, only 30% visited a nephrologist in 2013, compared with 91% who visited a PCP and 60% a cardiologist [46]. Among those patients with CKD stage 3, only 49% visited a nephrologist and even among those with CKD stage 4, only 67% visited a nephrologist the year following diagnosis, suggesting timely referral is low even among persons with insurance. Late referral of patients with CKD to a nephrologist is not restricted to the United States; it occurs in many countries [68]. |

**Supplemental Table 4. Clinical Practice Guidelines for Screening and Management of DKD**

| American Diabetes Association [15,23] | - Yearly measurement of UAE and eGFR in all patients with T2DM and in all patients with hypertension - Management   - Yearly UAE, eGFR, and serum K^+^ in all patients   - eGFR 45‒60 mL/min/1.73 m^2^: eGFR every  6 months, monitor electrolytes   - eGFR 30‒44 mL/min/1.73 m^2^: eGFR every  3 months, monitor electrolytes   - eGFR <30 mL/min/1.73 m^2^: referral to nephrologist |
| --- | --- |
| KDOQI [16] | - Yearly measurement of UAE and eGFR in all patients with T2DM |

DKD, diabetic kidney disease; eGFR, estimated glomerular filtration rate; K, potassium; KDOQI, Kidney Disease Outcomes Quality Initiative; T2DM, type 2 diabetes; UAE, urinary albumin excretion.

**References**

1. Centers for Disease Control and Prevention. National Diabetes Statistics Report, 2014 2014 [cited 2016 December 7]. Available from: <http://www.cdc.gov/diabetes/pubs/statsreport14/national-diabetes-report-web.pdf>.

2. Plantinga LC, Tuot DS, Powe NR. Awareness of chronic kidney disease among patients and providers. Adv Chronic Kidney Dis. 2010;17(3):225-36.

3. Retnakaran R, Cull CA, Thorne KI, Adler AI, Holman RR, Group US. Risk factors for renal dysfunction in type 2 diabetes: U.K. Prospective Diabetes Study 74. Diabetes. 2006;55(6):1832-9.

4. Bash LD, Selvin E, Steffes M, Coresh J, Astor BC. Poor glycemic control in diabetes and the risk of incident chronic kidney disease even in the absence of albuminuria and retinopathy: Atherosclerosis Risk in Communities (ARIC) Study. Arch Intern Med. 2008;168(22):2440-7.

5. UK Prospective Diabetes Study (UKPDS) Group. Intensive blood-glucose control with sulphonylureas or insulin compared with conventional treatment and risk of complications in patients with type 2 diabetes (UKPDS 33). Lancet. 1998;352(9131):837-53.

6. Zoungas S, Chalmers J, Ninomiya T, Li Q, Cooper ME, Colagiuri S, et al. Association of HbA1c levels with vascular complications and death in patients with type 2 diabetes: evidence of glycaemic thresholds. Diabetologia. 2012;55(3):636-43.

7. Wanner C, Inzucchi SE, Lachin JM, Fitchett D, von Eynatten M, Mattheus M, et al. Empagliflozin and progression of kidney disease in type 2 diabetes. N Engl J Med. 2016;375(4):323-34.

8. Heerspink HJ, Desai M, Jardine M, Balis D, Meininger G, Perkovic V. Canagliflozin slows progression of renal function decline independently of glycemic effects. J Am Soc Nephrol. 2016.

9. Mosenzon O, Leibowitz G, Bhatt DL, Cahn A, Hirshberg B, Wei C, et al. Effect of saxagliptin on renal outcomes in the SAVOR-TIMI 53 Trial. Diabetes Care. 2016.

10. Zinman B, Wanner C, Lachin JM, Fitchett D, Bluhmki E, Hantel S, et al. Empagliflozin, cardiovascular outcomes, and mortality in type 2 diabetes. N Engl J Med. 2015;373(22):2117-28.

11. Marso SP, Daniels GH, Brown-Frandsen K, Kristensen P, Mann JF, Nauck MA, et al. Liraglutide and cardiovascular outcomes in type 2 diabetes. N Engl J Med. 2016;375(4):311-22.

12. American Diabetes Association. 6. Glycemic Targets. Diabetes Care. 2017;40(Suppl 1):S48-S56.

13. National Kidney Foundation. KDOQI Clinical Practice Guideline for Diabetes and CKD: 2012 Update. Am J Kidney Dis. 2012;60(5):850-86.

14. Begun A, Icks A, Waldeyer R, Landwehr S, Koch M, Giani G. Identification of a multistate continuous-time nonhomogeneous Markov chain model for patients with decreased renal function. Med Decis Making. 2013;33(2):298-306.

15. American Diabetes Association. 10. Microvascular Complications and Foot Care. Diabetes Care. 2017;40(Suppl 1):S88-S98.

16. National Kidney Foundation. KDOQI Clinical Practice Guidelines and Clinical Practice Recommendations for Diabetes and Chronic Kidney Disease. Am J Kidney Dis. 2007;49(suppl 2):s1-s180.

17. Ali MK, Bullard KM, Saaddine JB, Cowie CC, Imperatore G, Gregg EW. Achievement of goals in U.S. diabetes care, 1999-2010. N Engl J Med. 2013;368(17):1613-24.

18. Stark Casagrande S, Fradkin JE, Saydah SH, Rust KF, Cowie CC. The prevalence of meeting A1C, blood pressure, and LDL goals among people with diabetes, 1988-2010. Diabetes Care. 2013;36(8):2271-9.

19. Parving HH, Lehnert H, Brochner-Mortensen J, Gomis R, Andersen S, Arner P, et al. The effect of irbesartan on the development of diabetic nephropathy in patients with type 2 diabetes. N Engl J Med. 2001;345(12):870-8.

20. Lewis EJ, Hunsicker LG, Clarke WR, Berl T, Pohl MA, Lewis JB, et al. Renoprotective effect of the angiotensin-receptor antagonist irbesartan in patients with nephropathy due to type 2 diabetes. N Engl J Med. 2001;345(12):851-60.

21. Brenner BM, Cooper ME, de Zeeuw D, Keane WF, Mitch WE, Parving HH, et al. Effects of losartan on renal and cardiovascular outcomes in patients with type 2 diabetes and nephropathy. N Engl J Med. 2001;345(12):861-9.

22. Lewis EJ, Hunsicker LG, Bain RP, Rohde RD. The effect of angiotensin-converting-enzyme inhibition on diabetic nephropathy. The Collaborative Study Group. N Engl J Med. 1993;329(20):1456-62.

23. Krauss RM. Lipids and lipoproteins in patients with type 2 diabetes. Diabetes Care. 2004;27(6):1496-504.

24. Morton J, Zoungas S, Li Q, Patel AA, Chalmers J, Woodward M, et al. Low HDL cholesterol and the risk of diabetic nephropathy and retinopathy: results of the ADVANCE study. Diabetes Care. 2012;35(11):2201-6.

25. Russo GT, De Cosmo S, Viazzi F, Pacilli A, Ceriello A, Genovese S, et al. Plasma triglycerides and HDL-C levels predict the development of diabetic kidney disease in subjects with type 2 diabetes: the AMD Annals Initiative. Diabetes Care. 2016;39(12):2278-87.

26. Abbate M, Zoja C, Remuzzi G. How does proteinuria cause progressive renal damage? J Am Soc Nephrol. 2006;17(11):2974-84.

27. KDOQI Clinical Practice Guidelines and Clinical Practice Recommendations for Diabetes and Chronic Kidney Disease. Am J Kidney Dis. 2007;49(suppl 2):s1-s180.

28. Astor BC, Matsushita K, Gansevoort RT, van der Velde M, Woodward M, Levey AS, et al. Lower estimated glomerular filtration rate and higher albuminuria are associated with mortality and end-stage renal disease. A collaborative meta-analysis of kidney disease population cohorts. Kidney Int. 2011;79(12):1331-40.

29. van der Velde M, Matsushita K, Coresh J, Astor BC, Woodward M, Levey A, et al. Lower estimated glomerular filtration rate and higher albuminuria are associated with all-cause and cardiovascular mortality. A collaborative meta-analysis of high-risk population cohorts. Kidney Int. 2011;79(12):1341-52.

30. Amin AP, Whaley-Connell AT, Li S, Chen SC, McCullough PA, Kosiborod MN, et al. The synergistic relationship between estimated GFR and microalbuminuria in predicting long-term progression to ESRD or death in patients with diabetes: results from the Kidney Early Evaluation Program (KEEP). Am J Kidney Dis. 2013;61(4 Suppl 2):S12-23.

31. de Zeeuw D, Remuzzi G, Parving HH, Keane WF, Zhang Z, Shahinfar S, et al. Proteinuria, a target for renoprotection in patients with type 2 diabetic nephropathy: lessons from RENAAL. Kidney Int. 2004;65(6):2309-20.

32. Hellemons ME, Persson F, Bakker SJ, Rossing P, Parving HH, De Zeeuw D, et al. Initial angiotensin receptor blockade-induced decrease in albuminuria is associated with long-term renal outcome in type 2 diabetic patients with microalbuminuria: a post hoc analysis of the IRMA-2 trial. Diabetes Care. 2011;34(9):2078-83.

33. Hu J, Yang S, Zhang A, Yang P, Cao X, Li X, et al. Abdominal obesity is more closely associated with diabetic kidney disease than general obesity. Diabetes Care. 2016;39(10):e179-80.

34. Kovesdy CP, Furth SL, Zoccali C, World Kidney Day Steering C. Obesity and kidney disease: hidden consequences of the epidemic. Am J Nephrol. 2017;45(3):283-91.

35. Bruce MA, Beech BM, Crook ED, Sims M, Griffith DM, Simpson SL, et al. Sex, weight status, and chronic kidney disease among African Americans: the Jackson Heart Study. J Investig Med. 2013;61(4):701-7.

36. Chen J, Muntner P, Hamm LL, Jones DW, Batuman V, Fonseca V, et al. The metabolic syndrome and chronic kidney disease in U.S. adults. Ann Intern Med. 2004;140(3):167-74.

37. KDIGO Clinical practice guideline for acute kidney injury. Kidney Int Suppl (2011). 2012;2(1):1-138.

38. Chawla LS, Eggers PW, Star RA, Kimmel PL. Acute kidney injury and chronic kidney disease as interconnected syndromes. N Engl J Med. 2014;371(1):58-66.

39. Heung M, Steffick DE, Zivin K, Gillespie BW, Banerjee T, Hsu CY, et al. Acute Kidney Injury Recovery Pattern and Subsequent Risk of CKD: An Analysis of Veterans Health Administration Data. Am J Kidney Dis. 2016;67(5):742-52.

40. Lo LJ, Go AS, Chertow GM, McCulloch CE, Fan D, Ordonez JD, et al. Dialysis-requiring acute renal failure increases the risk of progressive chronic kidney disease. Kidney Int. 2009;76(8):893-9.

41. Thakar CV, Christianson A, Himmelfarb J, Leonard AC. Acute kidney injury episodes and chronic kidney disease risk in diabetes mellitus. Clin J Am Soc Nephrol. 2011;6(11):2567-72.

42. De Cosmo S, Viazzi F, Pacilli A, Giorda C, Ceriello A, Gentile S, et al. Serum uric acid and risk of CKD in type 2 diabetes. Clin J Am Soc Nephrol. 2015;10(11):1921-9.

43. Hojs R, Ekart R, Bevc S, Hojs N. Markers of inflammation and oxidative stress in the development and progression of renal disease in diabetic patients. Nephron. 2016;133(3):159-62.

44. Shankar A, Sun L, Klein BE, Lee KE, Muntner P, Nieto FJ, et al. Markers of inflammation predict the long-term risk of developing chronic kidney disease: a population-based cohort study. Kidney Int. 2011;80(11):1231-8.

45. Schievink B, Kropelin T, Mulder S, Parving HH, Remuzzi G, Dwyer J, et al. Early renin-angiotensin system intervention is more beneficial than late intervention in delaying end-stage renal disease in patients with type 2 diabetes. Diabetes Obes Metab. 2016;18(1):64-71.

46. United States Renal Data System. 2016 USRDS annual data report: Epidemiology of kidney disease in the United States. National Institutes of Health, National Institute of Diabetes and Digestive and Kidney Diseases, Bethesda, MD, 2016. 2016 [cited 2016 December 7]. Available from: <https://www.usrds.org/adr.aspx>.

47. Kurella Tamura M, Anand S, Li S, Chen SC, Whaley-Connell AT, Stevens LA, et al. Comparison of CKD awareness in a screening population using the Modification of Diet in Renal Disease (MDRD) study and CKD Epidemiology Collaboration (CKD-EPI) equations. Am J Kidney Dis. 2011;57(3 Suppl 2):S17-23.

48. Plantinga LC, Boulware LE, Coresh J, Stevens LA, Miller ER, 3rd, Saran R, et al. Patient awareness of chronic kidney disease: trends and predictors. Arch Intern Med. 2008;168(20):2268-75.

49. Ene-Iordache B, Perico N, Bikbov B, Carminati S, Remuzzi A, Perna A, et al. Chronic kidney disease and cardiovascular risk in six regions of the world (ISN-KDDC): a cross-sectional study. Lancet Glob Health. 2016;4(5):e307-19.

50. Bruce MA, Beech BM, Crook ED, Sims M, Wyatt SB, Flessner MF, et al. Association of socioeconomic status and CKD among African Americans: the Jackson Heart Study. Am J Kidney Dis. 2010;55(6):1001-8.

51. Choi AI, Weekley CC, Chen SC, Li S, Tamura MK, Norris KC, et al. Association of educational attainment with chronic disease and mortality: the Kidney Early Evaluation Program (KEEP). Am J Kidney Dis. 2011;58(2):228-34.

52. Morton RL, Schlackow I, Staplin N, Gray A, Cass A, Haynes R, et al. Impact of educational attainment on health outcomes in moderate to severe CKD. Am J Kidney Dis. 2016;67(1):31-9.

53. Hall YN, Choi AI, Chertow GM, Bindman AB. Chronic kidney disease in the urban poor. Clin J Am Soc Nephrol. 2010;5(5):828-35.

54. Crews DC, McClellan WM, Shoham DA, Gao L, Warnock DG, Judd S, et al. Low income and albuminuria among REGARDS (Reasons for Geographic and Racial Differences in Stroke) study participants. Am J Kidney Dis. 2012;60(5):779-86.

55. Ricardo AC, Flessner MF, Eckfeldt JH, Eggers PW, Franceschini N, Go AS, et al. Prevalence and Correlates of CKD in Hispanics/Latinos in the United States. Clin J Am Soc Nephrol. 2015;10(10):1757-66.

56. Wright JA, Wallston KA, Elasy TA, Ikizler TA, Cavanaugh KL. Development and results of a kidney disease knowledge survey given to patients with CKD. Am J Kidney Dis. 2011;57(3):387-95.

57. Wright Nunes JA, Wallston KA, Eden SK, Shintani AK, Ikizler TA, Cavanaugh KL. Associations among perceived and objective disease knowledge and satisfaction with physician communication in patients with chronic kidney disease. Kidney Int. 2011;80(12):1344-51.

58. Jurkovitz CT, Li S, Norris KC, Saab G, Bomback AS, Whaley-Connell AT, et al. Association between lack of health insurance and risk of death and ESRD: results from the Kidney Early Evaluation Program (KEEP). Am J Kidney Dis. 2013;61(4 Suppl 2):S24-32.

59. Agrawal V, Jaar BG, Frisby XY, Chen SC, Qiu Y, Li S, et al. Access to health care among adults evaluated for CKD: findings from the Kidney Early Evaluation Program (KEEP). Am J Kidney Dis. 2012;59(3 Suppl 2):S5-15.

60. Szczech LA, Stewart RC, Su HL, DeLoskey RJ, Astor BC, Fox CH, et al. Primary care detection of chronic kidney disease in adults with type-2 diabetes: the ADD-CKD Study (awareness, detection and drug therapy in type 2 diabetes and chronic kidney disease). PLoS One. 2014;9(11):e110535.

61. Guessous I, McClellan W, Vupputuri S, Wasse H. Low documentation of chronic kidney disease among high-risk patients in a managed care population: a retrospective cohort study. BMC Nephrol. 2009;10:25.

62. Boulware LE, Troll MU, Jaar BG, Myers DI, Powe NR. Identification and referral of patients with progressive CKD: a national study. Am J Kidney Dis. 2006;48(2):192-204.

63. Kruse RL, Olsberg JE, Oliver DP, Shigaki CL, Vetter-Smith MJ, LeMaster JW. Patient-provider communication about diabetes self-care activities. Fam Med. 2013;45(5):319-22.

64. Smart NA, Dieberg G, Ladhani M, Titus T. Early referral to specialist nephrology services for preventing the progression to end-stage kidney disease. Cochrane Database Syst Rev. 2014(6):CD007333.

65. Singhal R, Hux JE, Alibhai SM, Oliver MJ. Inadequate predialysis care and mortality after initiation of renal replacement therapy. Kidney Int. 2014;86(2):399-406.

66. Gillespie BW, Morgenstern H, Hedgeman E, Tilea A, Scholz N, Shearon T, et al. Nephrology care prior to end-stage renal disease and outcomes among new ESRD patients in the USA. Clin Kidney J. 2015;8(6):772-80.

67. Nee R, Yuan CM, Hurst FP, Jindal RM, Agodoa LY, Abbott KC. Impact of poverty and race on pre-end-stage renaldisease care among dialysis patients in the United States. Clinical Kidney Journal. 2016:1-7.

68. Baer G, Lameire N, Van Biesen W. Late referral of patients with end-stage renal disease: an in-depth review and suggestions for further actions. NDT Plus. 2010;3(1):17-27.
